# Using behaviour change theory to train health workers on tobacco cessation support for tuberculosis patients: a mixed-methods study in Bangladesh, Nepal and Pakistan

**DOI:** 10.1186/s12913-019-3909-4

**Published:** 2019-01-25

**Authors:** Sahil Warsi, Helen Elsey, Melanie Boeckmann, Maryam Noor, Amina Khan, Deepa Barua, Shammi Nasreen, Samina Huque, Rumana Huque, Sudeepa Khanal, Prabin Shrestha, James Newell, Omara Dogar, Kamran Siddiqi, The TB & Tobacco consortium

**Affiliations:** 1 Leeds Institute of Health Sciences, Level 10 Worsley Building, Clarendon Way, Leeds, LS2 9NL UK; 2 Institute of General Practice, Addiction Research and Clinical Epidemiology Unit, Medical Faculty of the Heinrich-Heine-University Düsseldorf, Werdener Str. 4, 40227 Düsseldorf, Germany; 3 The Initiative, Orange Grove Farm, Banigala, Islamabad, Pakistan; 4 ARK Foundation, House B130, Road 21, New DOHS, Mohakhali, Dhaka, 1206 Bangladesh; 5 HERD International, P O Box Number: 24144, Thapathali 11, Kathmandu, Nepal; 6 The Hull York Medical School, University of York, York, YO10 5DD UK

**Keywords:** Health workers, Behaviour change, Tobacco cessation, Training Programme, LMICs

## Abstract

**Background:**

Low- and middle-income countries (LMICs) are disproportionately impacted by interacting epidemics of tuberculosis (TB) and tobacco consumption. Research indicates behavioural support delivered by health workers effectively promotes tobacco cessation. There is, however, a paucity of training to support LMIC health workers deliver effective tobacco cessation behavioural support. The TB and Tobacco Consortium undertook research in South Asia to understand factors affecting TB health workers’ delivery of tobacco cessation behavioural support, and subsequently developed a training package for LMICs.

**Methods:**

Using the “capability, opportunity, and motivation as determinants of behaviour” (COM-B) framework to understand any issues facing health worker delivery of behaviour support, we analysed 25 semi-structured interviews and one focus group discussion with TB health workers, facility in-charges, and national tuberculosis control programme (NTP) staff members in each country. Results were integrated with findings of an adapted COM-B questionnaire on health worker confidence in tobacco cessation support delivery, administered to 36 TB health workers. Based on findings, we designed a guide and training programme on tobacco cessation support for health workers.

**Results:**

Qualitative results highlighted gaps in the majority of health workers’ knowledge on tobacco cessation and TB and tobacco interaction, inadequate training on patient communication, insufficient resources and staff support, and NTPs’ non-prioritization of tobacco cessation in all three countries. Questionnaire results reiterated the knowledge deficits and low confidence in patient communication. Participants suggested strengthening knowledge, skills, and competence through training and professional incentives. Based on findings, we developed an interactive two-day training and TB health worker guide adaptable for LMICs, focusing on evidence of best practice on TB and tobacco cessation support, communication, and rapport building with patients.

**Conclusions:**

TB health workers are essential in addressing the dual burden of TB and tobacco faced by many LMICs. Factors affecting their delivery of tobacco cessation support can be identified using the COM-B framework, and include issues such as individuals’ knowledge and skills, as well as structural barriers like professional support through monitoring and supervision. While structural changes are needed to tackle the latter, we have developed an adaptable and engaging health worker training package to address the former that can be delivered in routine TB care.

**Trial registration:**

ISRCTN43811467.

**Electronic supplementary material:**

The online version of this article (10.1186/s12913-019-3909-4) contains supplementary material, which is available to authorized users.

## Background

The epidemics of tobacco consumption and tuberculosis (TB) interact synergistically to adversely impact health in low- and middle-income countries (LMICs) [1, 2]. Approximately 6 million new and relapse cases of pulmonary TB are notified annually worldwide, contributing to about 1 million TB deaths globally every year, with 81% of these occurring in LMICs, where smoking prevalence is also high [3, 4]. Smoking tobacco almost doubles the risk of acquiring TB infection and progressing from TB infection to TB disease, and negatively affects TB treatment outcomes, increasing the risk of dying from TB [5–7]. At the current rate of use, tobacco is projected to cause 18 million additional cases of TB and 40 million excess TB-related deaths between 2010 and 2050 [8].

Tobacco cessation strategies delivered by health workers, including pharmacological interventions and behaviour support methods, have been shown to be effective in helping people stop smoking [9–13]. However, research from South and East Asia indicates that implementing tobacco cessation programmes in LMICs is impeded by inadequate training and staff support for health workers, their consequent lack of knowledge of the effects of tobacco on health, and their lack of experience in providing tobacco cessation assistance [14–17]. Research highlights the challenges of including such behavioural support interventions in routine practice [18, 19] and there is little guidance for implementing tobacco cessation programmes in LMICs, particularly within National TB control Programmes (NTPs).

To address this gap, the TB and Tobacco Consortium – an EU-funded consortium of nine institutions in seven countries – is undertaking a four-year project (2016 to 2019) in Bangladesh, Nepal, and Pakistan that aims to investigate how interventions designed to encourage tobacco cessation can be integrated into routine NTP practice. All three countries have a high burden of TB and tobacco. WHO estimate an incidence rate of 221 (161–291) per 100,000 Bangladesh, 152 (134–172) per 100,000 in Nepal and 267 (189–357) per 100,000 Pakistan [20]. All three countries have a higher prevalence of both tobacco use and TB among men than women. According to WHO’s 2015 estimates, in Bangladesh the prevalence of smoking any tobacco product among males aged 15 years or more was 39.8% (30.6–50.1%) and 0.7% [0.4–1.0%] among women; in Nepal 37.1%([29.0–47.3%)among men and 11.1% (7.8–14.4%); in Pakistan 41.9% (29.7–57.3%) among men and 3.0% (1.8–4.2%) among women [21].

Working with real-world constraints in national TB programmes, one of the research aims was to understand issues influencing TB health workers’ delivery of behavioural support. These are health workers whose main work, sometimes exclusively, is with people diagnosed with TB. They are responsible for supporting patients to follow the six months of medication required in the ‘Directly Observed Treatment Short-course’ (DOTS) regime. The exact role and title of these health workers differs between the NTPs in Bangladesh, Nepal and Pakistan; throughout this paper they are referred to as ‘health workers’. To address our aim, we employed the “capability, opportunity, and motivation as determinants of behaviour” (COM-B) [22] framework in research design and analysis to assist in identifying key issues affecting health worker delivery of behavioural support. The COM-B framework, which was created from a systematic review of nineteen existing behaviour change theories, considers behaviour change as premised on individuals’ capabilities, opportunities and motivations to perform a behaviour [22, 23] This paper presents our COM-B analysis of factors affecting TB health workers’ delivery of behavioural support and how this analysis influenced the training package we subsequently developed.

## Methods

We conducted mixed-methods research with a convergent research design, where qualitative and quantitative data were collected and analysed separately before integration [24]. Research was primarily qualitative, with supplementary insight provided through a quantitative health worker questionnaire [25].

### Quantitative component

Quantitative data on TB health workers’ confidence in delivering tobacco cessation support was obtained using an interviewer-facilitated questionnaire, adapted from a UK National Centre for Smoking Cessation and Training (NCSCT) questionnaire (see Additional file 1).

Researchers used convenience sampling to approach at least one TB health worker from all 38 facilities involved in the trial in Bangladesh, Nepal, and Pakistan. The questionnaire was completed by TB health workers across This information was collected for guidance rather than to be representative, so no sample size calculations were done.

Developed under the COM-B framework, the questionnaire asks respondents to rate their confidence on supporting aspects of patients’ COM-B through providing tobacco cessation behavioural support using a scale of 1–5, where 1 is ‘not confident’ and 5 is ‘very confident’. In terms of health worker COM-B for delivering the intervention the questionnaire provides greatest insight into the capability (C), but may also reflect motivation and opportunity.

The adapted NCSCT questionnaire (Additional file 1) consisted of fourteen questions, and was translated into the local languages, back-translated into English, and then checked by the UK team. The questionnaire was adapted following piloting with three health workers from among the two case-study sites in each country and in close consultation with partners to identify issues pertinent to the context. Pre-tests elicited feedback on the clarity, acceptability, and relevance of each question; sought suggestions on alternative wording, phrasing, or additional questions; and invited any additional observations. For example, in the original NCSCT questionnaire there is only one gender-neutral question regarding rapport building with patients. Given challenges in communication between health and patients of the opposite sex, this question was split into two to address health worker rapport-building across genders. Further adaptations included the removal of questions relating to carbon monoxide (CO) measurement as this is not available in routine care in any of the three countries. In addition questions were added to assess any existing cessation support offered in the health worker’s own clinic and to questions to for the health worker to self-report their own tobacco use.

The results from the piloted questionnaires were not used in the analysis due to the alterations made following piloting.

### Qualitative component

Qualitative information was collected to understand a range of issues affecting the integration of tobacco cessation within NTP practice at the level of individuals, facilities and national institutions. To understand the wider context of tobacco cessation and TB control and how this related to the potential for health workers to deliver cessation, semi-structured interviews (SSIs) were conducted with health workers, TB facility in-charges (who are clinic managers), district and national level NTP staff members, and, where possible, national level stake-holders involved in tobacco control. We kept some flexibility to allow researchers to use focus group discussions rather than SSIs where several health workers all wished to be interviewed together. Where health workers were at the same level in the hierarchy this was felt to be appropriate. In light of this, one focus group with health workers in Bangladesh was conducted.

Qualitative data collection was undertaken primarily at two case-study sites in each country. The sites were identified in collaboration with research partners, purposively selected from among the 38 facilities in Bangladesh, Nepal, and Pakistan that were part of the wider project. The case-study sites were chosen to reflect different key characteristics of facilities within each country based on the most important dimensions of difference in each country. In Bangladesh, an urban NGO-run clinic and a rural government centre were selected as TB services are delivered through government and NGO facilities and the urban and rural contexts are very different. An urban, NGO-run, national referral centre and a smaller, urban, government-run clinic were selected in Nepal, given the differences between the large referral centres and smaller clinics in the country. The urban/rural context and TB centre size are particular issues in Pakistan, so a large, urban, government-run tertiary hospital and a rural, government-run hospital were selected.

The appropriateness of the sample size for our qualitative data collection can be justified using the domains of ‘information power’ identified by Malterud et al. (2015) [26]. Firstly, the study aim was narrow in terms of addressing the delivery of tobacco cessation behaviour change to people with TB, rather than any aspect of patient behaviour change. Secondly, the sample was relatively specific as it focused on TB health workers, although the fact the health workers were based in different types of health facilities (NGO or government, urban or rural) and in different countries, necessitated more participants. The focus on two case-study sites in each country helped address this dimension of information power. Thirdly, COM-B was used as a well-established theory of aspects affecting health worker behaviour, and a modest number of participants were required considering this domain. Fourthly, the quality of the dialogue, particularly with health workers, facility in-charges, and NTP staff was good, with interviews lasting up to one hour and providing detailed information. Finally, the analysis strategy employed looked across cases, both in terms of location and type of participant (NTP staff, in-charge or health worker), indicating a larger sample was required. Considering these different dimensions of information power, we consider that our sample size for the qualitative element was sufficient to provide useful insights into the COM-B of health workers to deliver tobacco cessation.

Researchers experienced in mixed-method research undertook data collection in each country. All researchers possessed qualifications in public health or social science research and were provided with supplemental training on using the FGD and SSI guides by the UK team before data collection.

The UK team worked with partners in Nepal, Bangladesh and Pakistan to prepare FGD and SSI guides in English and checked national research partners’ translations into Nepali, Bengali, or Urdu for clarity and congruence with the English prototypes. SSI guides for health workers (Additional file 2) and facility in-charges (Additional file 3) enquired into health workers’ capabilities, opportunities, and motivations to provide tobacco cessation, as well as perceptions of patient tobacco use. The questions under each of the COM categories drew on learning from our previous research on implementing behaviour support in primary care in Nepal [15] and among respiratory patients as part of a randomised controlled trial in Pakistan [12]. Additional open-ended questions were included to ensure that other issues not identified in previous research would also emerge. The SSI guides for district- and central-level NTP staff (Additional files 4 and 5) focussed on national-level commitment to tobacco cessation and perceived barriers or facilitators to implementing tobacco cessation behavioural support within NTP practice at different organisational levels.

SSIs were conducted with TB health workers and facility in-charges from case-study sites, with district NTP staff members from case-study site districts, and with heads of the NTPs. SSI participants were approached by researchers either by email or phone, and SSIs were conducted at participants’ places of work.

Interviewers were given training on using the FGD and SSI guides by the UK team ahead of data collection. Qualitative research activities were conducted in participants’ local languages and lasted between 45 and 60 min. They were audio-recorded, transcribed and translated into English by national partner researchers. Initial transcripts were reviewed by the UK team to ensure verbatim transcription and standardization. Table 1 below provides details of the participants and methods used.

**Table 1:** Methods and participants

	Bangladesh	Nepal	Pakistan	Total
Semi-Structured Interviews				
National Tobacco Control Staff	–	–	*n* = 1 (1 M)	*n* = 1 (1 M)
Central Level NTP Staff	*n* = 1 (1 M)	–	*n* = 1 (1 M)	*n* = 2 (2 M)
District Level NTP Staff	*n* = 1 (1 M)	*n* = 2 (2 M)	*n* = 2 (1F, 1 M)	*n* = 5 (1F, 4 M)
Facility In-charges	*n* = 2 (1 F, 1 M)	*n* = 3 (3 F)	*n* = 2 (2 M)	*n* = 7 (4F, 3 M)
TB Health Workers	*n* = 4 (4F)	*n* = 3 (3F)	*n* = 3 (2F, 1 M)	*n* = 10 (9F, 1 M)
Total				*n* = 25 (14F, 11 M)
Focus Group Discussions				
TB Health Workers	*n* = 1 (2F, 2 M)	–	–	*n* = 1 (2F, 2 M)
Questionnaire				
TB Health Workers	*n* = 10 (2F, 8 M)	*n* = 14 (11F, 3 M)	*n* = 10 (5F, 5 M)	*n* = 34 (18F, 16 M)
Facility In-Charges	–	*n* = 2 (2F)	–	*n* = 2 (2F)
Total				*n* = 36 (20F, 16 M)

*M* male and *F* female

### Analysis

Analysis of qualitative data in the first year was conducted in two rounds using NVivo software. Only data from the second round is considered in this paper. The first round focused specifically on informing development of the behavioural support materials that would be used for behavioural support, through considering patient and health worker responses to the behavioural support materials. The second round focussed on the COM-B analysis of health worker delivery of tobacco cessation behavioural support.

To analyse qualitative data, a coding scheme was developed to look at TB health workers’ capabilities, opportunities and motivations to provide tobacco cessation behavioural support, both self-reported by health workers and perceived by other research participants. Table 2 illustrates how capabilities, opportunities and motivations were understood for coding purposes. The coding scheme was tested on three transcripts by SW, HE, MB, and MN to establish a standardised coding method. Each SSI and FGD transcript was coded deductively by one of the researchers and then checked and re-coded by another.

**Table 2:** COM-B component description with tobacco cessation assistance examples

COM-B Model Component		Definition	Example
Capability	Physical	Physical skill	Having the ability to deliver tobacco cessation support with a flipbook
	Psychological	Capacity to engage in necessary thought processes – comprehension/reasoning	Having appropriate knowledge of TB or tobacco to provide cessation support
Opportunity	Physical	Opportunity afforded by environment	Having a suitable location for cessation counselling
	Social	Opportunity afforded by the cultural milieu dictating how one thinks about things, e.g. the words/concepts that make up language	Feeling able to talk about tobacco use with women as well as men, regardless of cultural taboos
Motivation	Reflective	Reflective processes involving evaluations and plans	Reflecting on interaction with patients and identifying ways to deliver cessation messages so they are respond to the realities of patients’ lives.
	Automatic	Automatic processes involving emotions and impulses arising from associative learning and/or innate dispositions	Wanting to deliver tobacco cessation support because as a health worker, one should help people become healthy

Adapted from Atkins 2013 [62]

All coded transcripts were combined into a single NVivo file, and a framework matrix produced for each country, exploring each COM-B code by respondent. Each matrix was then thematically organised by hand [27]. Thematic comparisons were subsequently made across countries and across different participant groups by gender, patient age and education level, and by health worker occupational level. A narrative was prepared on the cross-country findings organized around health worker capabilities, opportunities, and motivations to provide tobacco cessation behavioural support.

Results of the adapted NCSCT questionnaire were input into an MS Excel worksheet by country. Mean responses to each question were calculated for each country and then across all countries. Responses were also analysed by respondents’ sex. This analysis of questionnaire results was compared with qualitative findings to identify any similarities or differences.

The quantitative results were explored within the qualitative findings by searching for sections of text that might confirm, disconfirm or shed further light on the quantitative results [28].

The findings from the quantitative and qualitative work informed the intervention materials, health worker training programme and health worker guide on tobacco cessation behavioural support. Specifically, the quantitative survey findings identified gaps in knowledge and confidence which primarily informed the content of the training programme and the text on the back of the flipbook for health workers to use during the behaviour support session with patients. The qualitative findings helped inform all aspects of the intervention materials and training, and also provided insights into the need for systems changes to support implementation of the intervention. In each country context, our teams were able to work with their NTPs to explore ways to address these health system challenges. The findings below highlight the elements relating to health worker behaviour that informed the material development and training.

## Results

Between June and September 2016, we conducted 25 SSIs and 12 FGDs across Bangladesh, Nepal, and Pakistan, and administered the adapted NCSCT questionnaire to 36 TB health workers. Details on research methods and participant breakdown by sex are provided in Table 1.

All planned activities were conducted, with the exception of a SSI with a central level NTP staff member in Nepal. While there was no explicit refusal, the participant refrained from agreeing to a date to conduct the SSI. An additional FGD with TB health workers was conducted in Bangladesh, using the FGD guide, to receive feedback on behavioural support materials prior to finalization.

Questionnaires were completed by at least one health worker in each of the 38 sites except five sites in Bangladesh, one in Nepal, and one in Pakistan. Logistical difficulties precluded administering the questionnaire at these sites. Two facility in-charges in Nepal, both women, also completed the questionnaire as their duties included providing TB support to patients that would involve tobacco cessation behavioural support.

### Qualitative results

Issues highlighted by research participants that affect TB health workers’ capabilities, opportunities, and motivations to deliver tobacco cessation behavioural support included a lack of knowledge and inadequate patient communication skills, a lack of resources and staff support for health workers, and low prioritization of tobacco cessation within NTPs. The primary suggestions offered by participants to increase health worker abilities were to provide training and professional incentives such as staff support or additional payments to conduct behavioural support.

### Health worker capability

Health workers’ SSI responses showed their clear understanding that tobacco use was harmful for TB patients. While some health workers reported providing patients with information or counselling on quitting tobacco use, in each country they reported that there was no standardized tobacco cessation information provided to all TB patients, or any means to report or record patient tobacco use. There was also a lack of awareness that sudden cessation is more effective than slow withdrawal [29], a typical response being:

“Normally we guide patients to maintain a good and healthy diet and, if they are smoking, to quit tobacco – quit it slowly but quit it as it is not good for one’s health. We tell them that you should stop smoking slowly.” (Pakistan, Health Worker 1)

Health workers across all three countries indicated a desire for training on tobacco and TB, motivated in part by patients sometimes asking about tobacco cessation assistance. This desire was raised by participants at all levels. One health worker stated:

“We also don’t know much about [tobacco]. There should be materials for counselling … there is not much training regarding counselling on smoking … The main [recommendation] is training.” (Nepal, Health Worker 1)

Health workers across all countries reported patients, especially women and young people, being loath to talk about tobacco use. Some expressed confidence in being able to gauge patient tobacco use by appearance. A health worker in Nepal stated: “When they come close, I know whether they smoke or not by the colour of their lips, their gums, or smell.” (Nepal, Health Worker 2) Another added, however, that training was required on how to counsel patients:

“We have not been trained on counselling… If we [health workers] had special counselling training, only then would we know [how to counsel properly].” (Nepal, Health Worker 1)

The need for effective health worker communication with patients was raised by all participants. A Pakistani facility in-charge stated that in order for proper behavioural support to be provided, health workers would need to have “good communication skills … then they will be able to [provide] counselling” (Pakistan, Facility In-Charge 1).

### Opportunity

Participants at facility, district and national levels all emphasised how health worker opportunities to provide behavioural support were affected by resources limitations and particularly the interrelated issues of high patient load and a dearth of time. Health workers reported the time-consuming process of how they manually record information on patients, which is only later stored electronically. A Pakistani health worker clarified:

“… we don’t have many facilities … follow-ups, record maintenance, everything is done manually. There are five registers for one year, one is only for registration, so already it is difficult for us to maintain, if [behavioural support] is added to our work, it will be difficult for us to manage.” (Pakistan, Health Worker 1)

A Bangladeshi health worker further explained:

“Generally, we do not face any problem except time pressure. Sometimes, due to high workloads or pressures, our work day ends at five or six pm instead of four.” (Bangladesh, Health Worker 1)

Health workers stressed how this affected the opportunity for providing behavioural support. One respondent (Pakistani Health Worker 2) was clear that it takes “maximum 5 minutes in TB counselling with a patient … [but with behavioural support] we will require 30 minutes per patient.” A Bangladeshi facility in-charge emphasized that

“… we have around 250 patients in our current load, and if we have to provide tobacco cessation support to around 100 patients along with the DOTs support to all patients then it will be an extra workload for us.” (Bangladesh, Facility In-Charge 1)

While Pakistani and Bangladeshi health workers framed the problem of patient load as connected to having too many patients, a health worker in Nepal suggested the issue was also related to an insufficient number of health workers. “We need manpower,” she explained, “in some places there is no manpower… you have to consider this.” (Nepal, Health Worker 1).

At an institutional level, research participants (facility in-charges and health workers) raised the issue of a lack of sufficient staff support through supervision and monitoring, leading to an atmosphere where tobacco cessation support is not something considered or discussed professionally. SSI participants in all countries described how no supervision or monitoring was provided to health workers, other than collecting statistics on TB patient outcomes. A Pakistani district-level NTP staff member suggested this was an issue of NTP resources, explaining

“There is no one for monitoring … we check [health workers’] registers and records … [to see] recordkeeping is going fine and we take feedback from the medical superintendent ...” (Pakistan, District NTP Staff 1)

At national policy level, health workers as well as district- and central-level NTP staff members, across all countries, raised the lack of NTP prioritisation of tobacco cessation, and consequent absence of the issue from their professional context, as reducing opportunity for tobacco cessation behavioural support delivery to TB patients. Central- and district-level NTP staff referred to priorities including TB outcomes, HIV, and MDR-TB, but indicated tobacco was not on the agenda. They felt, however, that tobacco should be addressed. A Nepali district-level staff member remarked:

“Priority should be given [to tobacco]. But it is not being done at present. Under National Tuberculosis Programme, the [WHO Practical Approach to Lung Health] PAL programme was introduced but could not be implemented effectively. Now, it is almost extinct ... the NTP should bring the TB programme and tobacco control together.” (Nepal, District NTP Staff 2)

### Motivation

Pakistani and Nepali health workers’ statements that they provided some information to patients on tobacco and smoking of their own accord is an expression of their ‘automatic motivation’ to provide tobacco cessation behavioural support. Both Bangladeshi health workers also indicated a vocational motivation to provide patients with such support. One of the health workers enthusiastically stated that

“We are working for TB patients’ betterment ... my goal and your goal [of providing tobacco cessation support] … will work together to bring better outcomes for patients.” (Bangladesh, Health Worker 1)

All participants indicated that the lack of resources and professional support would also affect health worker motivation to provide tobacco cessation behavioural support. The two main issues raised were insufficient work space and extra workloads. Professional incentives, e.g. training, adequate and specified work space, fixed workloads, and financial assistance, were the main suggestions proposed to address factors affecting health worker motivation.

Health workers described an interrelated set of issues negatively impacting health worker motivation. These related to inadequate working conditions including a lack of space or insufficient space, delegation of work to them in excess of their roles, and a lack of professional support or guidance through monitoring or supervision. A Nepali health worker suggested:

“We need record reporting registers [for tobacco use] or some cards. If we have such cards, we can report accordingly. If there is recording and reporting, work will be done properly … We also need training. Next, there should be supervision and monitoring … [and] proper management. [Laughing] Things related to paying also need to be done.” (Nepal, Health Worker 1)

In Pakistan, a district-level NTP staff member described the issue in detail, explaining that:

"[A]ll our [health workers] are not limited to the TB programme. They are nursing staff and their duties are rotational … If you release them from other duties, they will be focussed on TB and tobacco cessation, it will motivate them to not have extra evening and morning duties and just have fixed morning [TB] duty hours …

If you are adding this tobacco cessation job to their duties, first … strengthen them. They should have an office; their designation should be mentioned outside their office. If they do not feel good, their self-esteem will not be boosted and they won’t be able to perform tasks. Monetary incentives can be helpful for motivation, or a proper conducive environment and proper system can also be motivating. Along with this, they should have specific duties." (Pakistan, District Staff 1)

While Bangladeshi and Nepali SSI participants obliquely suggested financial incentives would be required to undertake any additional reporting on tobacco and delivery of tobacco cessation behavioural support, Pakistani participants at all levels were more direct:

“… for proper and smooth work, financial assistance and certificates should be awarded, that is moral and financial support … because then work will be done with interest … in the proper way …” (Pakistan, Health Worker 1)

Training and certification were also mentioned by some health workers as important for motivation.

### Quantitative results

**Fig. 1:**
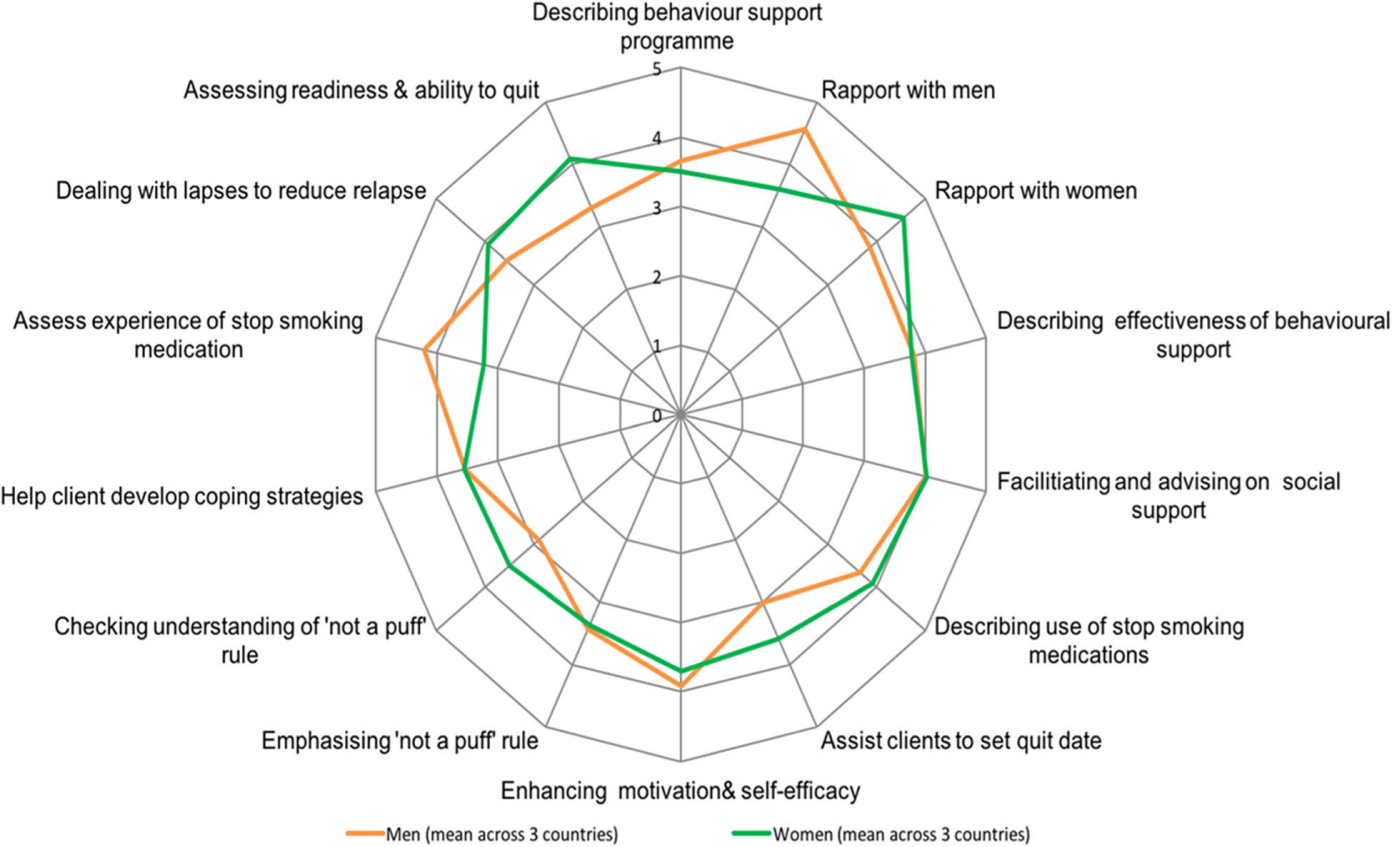
Mean questionnaire responses across Bangladesh, Nepal, and Pakistan

Responses to the questionnaire were largely consistent across all countries, with mean responses for each question falling between moderately and highly confident (Fig. 1). Echoing qualitative findings, respondents indicated lower confidence in assisting with setting a quit date and emphasising and checking patients’ understanding of sudden cessation. While health worker confidence in delivering tobacco cessation support hovered above moderately confident across all sites, there were two exceptions. Nepali male health workers indicated much lower confidence (mean of 2.0) in checking patients’ understanding of sudden cessation than others. Pakistani female health workers expressed much higher confidence than others in describing the use of stop smoking medication to patients (mean of 4.8). Both male and female health workers felt less confident in engaging with patients of the opposite sex, particularly in Pakistan (Table 3), reflecting qualitative findings on health workers’ interaction with patients.

**Table 3:** Confidence of health workers to deliver elements of COM-B to patients during tobacco cessation counselling by country and sex of health worker

	Mean Responses by Health Worker Sex (Response Scale: 1 = Not Confident, 3 = Moderately Confident, 5 = Highly Confident)						Overall
	Bangladesh		Nepal		Pakistan		
	Women	Men	Women	Men	Women	Men	
Describing behavioural support programme	3.5	3.9	3.8	3.7	3.2	3.4	3.6
Building rapport (men)	4.0	4.5	4.0	5.0	2.8	4.2	4.1
Building rapport (women)	4.5	3.5	4.4	4.7	4.8	3.4	4.2
Describing behavioural support effectiveness	4.0	3.6	3.5	3.7	3.8	4.2	3.8
Facilitating/ advising on social support	4.0	4.3	4.1	4.3	4.0	3.4	4.0
Describe stop smoking medication use	3.5	3.9	3.5	3.7	4.8	3.4	3.8
Assist in setting quit date	4.0	3.3	3.2	2.3	3.6	3.4	3.3
Enhancing motivation/ self-efficacy	3.0	4.4	3.9	3.3	4.2	4.0	3.8
Emphasising sudden cessation	3.5	3.1	2.8	4.0	3.8	3.2	3.4
Check sudden cessation understanding	3.5	3.5	3.2	2.0	3.8	3.2	3.2
Assist in developing coping strategies	3.5	3.6	3.4	3.0	3.8	4.0	3.5
Assess stop smoking medication experience	3.0	3.9	3.3	4.3	3.4	4.4	3.7
Reducing/dealing with lapses	3.5	4.0	3.9	3.7	4.4	3.0	3.7
Assess readiness/ ability to quit	4.5	3.5	3.4	3.0	4.4	3.4	3.7

### Training package development

**Fig. 2:**
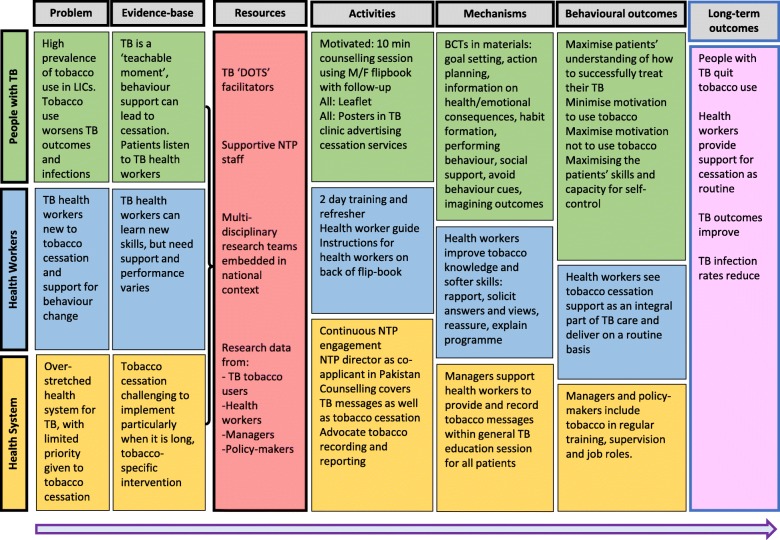
Theory of change to address health worker COM-B for behavioural support delivery

Combining the results of the qualitative and quantitative research, we identified a set of key issues affecting TB health worker COM-B to provide tobacco cessation behavioural support. With the exception of some structural issues such as a reported dearth of resources and lack of professional support, the key issues affecting health worker COM-B were related to health workers’ knowledge and skills. We developed a theory of change (Fig. 2) based on these issues, which we used to create a two-day TB health worker training programme and a health worker guide. The theory of change also highlights the health systems changes required to normalise the tobacco cessation intervention within routine care. Specifically, these include the inclusion of tobacco cessation within NTP policy and guidelines, revisions to routine TB reporting forms to include patient tobacco-use status, quit advice and quit outcome and inclusion of tobacco cessation within all NTP training (see Table 4). These health systems changes will do much to provide the opportunity for health workers to deliver cessation. Such national changes require detailed engagement with NTPs in each country and this forms another component of the wider study but is beyond the scope of this paper.

**Table 4:** Addressing Health Worker COM-B through training

Primary COM-B area addressed	Day One	Day Two
Motivation	Knowledge on TB and Tobacco interaction – current evidence, including of increased likelihood of quit due to HW support	Feedback from peers and trainers.
Opportunity	Introduction to all materials	Health workers consider their own facility to identify opportunities for cessation within patient flow
Capability	Role plays to practice delivering the key messages in the materials, to build communication skills and reiterate knowledge	Role plays to deal with ‘tricky’ situations and different types of patients (male/female/low literacy/with relatives present/unwilling to share tobacco use status)

We used an initial first round of analysis of the data (which also included focus groups with patients not reported here) to adapt and develop country-specific behavioural support materials including a flipbook, poster and leaflet for use with patients during consultations, and a health worker guide on behavioural support delivery to be distributed to all training participants. The guide addresses health worker skills and knowledge by providing clear and concise text which references the evidence underpinning the approach to tobacco cessation and the associations between tobacco use and TB. The guide is designed to increase health worker motivation by suggesting ways to integrate and deliver behavioural support in routine practice. Using text and images from the behavioural support materials, the guide provides an overview of:

i. The flipbook and leaflet with tips on delivery and patient engagement
ii. Global facts on tobacco, evidence-base on TB and tobacco interaction, and dangers of smokeless tobacco
iii. Information on nicotine and difficulties in quitting tobacco
iv. Tips for active listening and examples on rapport building skills with different kinds of patients
v. How to undertake follow-up with patients on tobacco cessation efforts.

The training programme covers all the topics above with scenarios to use in role-plays which allow health workers to practice the delivery of cessation advice.

## Discussion

Employing the COM-B framework to structure data collection and analysis allowed us to understand factors affecting TB health workers’ delivery of tobacco cessation behavioural support in Bangladesh, Nepal, and Pakistan within a limited timeframe, and is repeatable in other implementation research settings in LMICs. Health workers’ reported confidence to deliver cessation support was higher than the research team expected, given their current lack of training in this area. We hypothesise that this may be due to a tendency to overestimate skills in this area. Based on our findings, we developed a health worker training package consisting of a two-day training programme and a health worker guide. While training TB health workers could address key barriers in tobacco cessation integration into routine care, wider health system approaches will also be needed.

Across the three countries, we found health workers recognised the need to deliver tobacco cessation behavioural support to patients. However, they suggested their ability to do so was hindered by a set of factors including a lack of knowledge about tobacco and TB interaction, low understanding of tobacco cessation, and poor patient communication skills. Additional barriers they identified were an institutional lack of resources (insufficient space, high patient load, no reporting/recording of tobacco, overwork) and an absence of professional support through monitoring and evaluation. The two main suggestions participants offered in addressing these barriers were providing health worker training, and addressing health worker support through provision of supervision and financial incentives.

Our findings echo those from studies in Australia and the UK that have used the COM-B framework to respectively develop health worker training on tobacco cessation and physical activity advice for patients. These studies also found a need to address the health worker knowledge base, confidence, and competence to engage and communicate with patients. They also identified the need to build a supportive environment in the workplace by addressing the dearth of time and resources available to health workers and prioritisation of the activity within services [30, 31]. The suggestion by our participants to strengthen supervision to increase motivation was not highlighted in these studies, but is part of creating a supportive professional environment to integrate behavioural support into routine practice.

The findings on health worker delivery of tobacco cessation behavioural support are also consistent with research in other LMICs in Asia on integrating tobacco cessation behavioural support into public health systems, and TB control in particular. This research reveals the lack of integration of tobacco cessation into health worker training [14–17]. The gender and smoking status of the health worker is also found to influence the extent to which cessation is delivered, with female health workers who do not smoke more likely to provide cessation advice than male health workers who do or do not smoke [16]. These studies recommend adapting cessation delivery training to health worker contexts, as well as the instituting of regular recording, reporting, supervision and leadership on tobacco cessation for health workers [14–17]. Despite evidence that training health workers in tobacco cessation behavioural support is effective in increasing cessation among patients [32, 33], health programmes in LMICs are reportedly less likely to do so than in other countries. Reasons cited are policy-level perceptions of extra monetary and human resource investments, the absence of cost-effective models for training health workers on tobacco cessation, and a lack of appropriate trainers and training materials [34, 35].

Given that our theory of change has been developed based on empirical work in three country contexts, our findings are likely to be of relevance to other LMIC contexts, particularly in Asia. The training package we have developed could be adapted for use in other LMICs. In addition to providing evidence-based information on TB and tobacco interaction, it trains health workers on behavioural support skills including positive rapport-building with patients. Effective communication with patients, through demonstrating emotional understanding and encouraging patients to open up, has been shown to positively affect patient outcomes [36–39]. It also uses low-resource techniques such as practice in rehearsing behavioural support skills with feedback, and developing patient flow diagrams to adapt behavioural support methods to facility contexts, which have been shown to be effective in enabling health workers to integrate new practices and increase quality of healthcare [40, 41].

Training health workers in new skills such as behavioural support has been shown to improve their confidence and delivery of quality of care [42, 43]. Integrating such new skills does however have costs for health workers including increased workloads and time pressure, making implementation challenging in the long term [37]. While training can assist health workers in accommodating some changes, research in LMICs highlights a primary barrier to long-term implementation lies in national systems’ understaffing and low health worker support. These constraints not only diminish health workers’ abilities to provide behavioural support, as our findings indicate, but can also foster a general culture leading health workers to adopt a directive approach to counselling with less patient engagement [44–46].

Participants in our research suggested the provision of financial incentives and increased support through monitoring and supervision as ways to address health system-level barriers. Evidence from LMICs demonstrates low salaries can lead to decreased motivation of health workers and burn-out through the need for supplemental employment [47–50]. However, research on the effectiveness of providing financial incentives is mixed, with indications that inadequate institutional infrastructure and support diminishes any effectiveness of financial incentives [51–53]. Studies further indicate that when provided, financial incentives must be coupled with non-financial incentives to effectively increase health worker motivation, with investment in human resources being an effective way of increasing health worker performance [54, 55].

Investing in human resources can include an array of actions. Researchers in low-resource settings advocate providing health workers with supervision as the most effective means of improving their performance by increasing job satisfaction and motivation [56], echoing findings from our study. While supervision plans made at the policy level that are in line with international standards but do not take local context into account might be unrealistic and not cost-effective, research literature suggests low-cost solutions to strengthening supervisors can be employed in LMICs through efforts such as local training in management or team building exercises [56, 57]. Research on LMIC health systems indicates, however, that multi-pronged interventions, incorporating training and supervision, that address multiple determinants of health worker performance, are most likely to be effective in these contexts [41, 56, 58, 59].

### Strengths and limitations

The design of the study has several strengths including the use of multiple data sources (health workers, policy makers and patients), multiple methods (focus groups, interviews and questionnaire) and drawing on the theoretical framework of COM-B to guide data collection and analysis [60]. As described in the methods, we believe the sample provided sufficient information power [27] to answer our research question. A further aid to the trustworthiness of the data was the use of multiple analysts to develop a coding framework and to code the qualitative data, which enabled the team to discuss which COM-B domain was most appropriate for each issue raised by respondents [61].

While the qualitative approach was not designed to provide findings that can be uncritically generalised to other sites, the variation in facilitaties (urban/rural, NGO/government) allow for considerable transferability. However, it should be noted that remote rural sites, for example the mountain zones of Nepal and Pakistan were not included as case studies and that this may limit the transferability of these findings to remote rural settings.

## Conclusion

The dual synergistic burden of TB and tobacco faced by LMICs calls for an integrated approach to tackling tobacco use among TB patients. TB health workers play a key role in addressing this issue. Factors identifying health worker skills and abilities to provide services such as tobacco cessation behavioural support can be quickly identified using the COM-B framework. These are likely to include issues at the health worker level, such as individuals’ knowledge and skills, as well as structural barriers such as inadequate professional support through monitoring and supervision. While changes to the health system are needed to tackle the latter, the former can be addressed through developing robust and engaging health worker training that fits within routine training within the health system.

## Additional files

Additional file 1: COM-B Questionnaire –English Version. English version of adapted COM-B Questionnaire (DOCX 35 kb)

Additional file 2: Health Worker Semi-Structured Interview Guide. English version of health worker SSI guide. (PDF 47 kb)

Additional file 3: Facility In-Charge Semi-Structured Interview Guide. English version of facility in-charge SSI guide. (PDF 47 kb)

Additional file 4: District-Level NTP Staff Semi-Structured Interview Guide. English version of district-level TB staff SSI guide (PDF 47 kb)

Additional file 5: Central-Level NTP and National-Level Stakeholders Semi-Structured Interview Guide. English version of central-level TB staff SSI guide. (PDF 46 kb)

## Abbreviations

COM-B: Capability, Opportunity, and Motivation as Determinants of Behaviour
FGD: Focus Group Discussion
LMIC: Low- and Middle-Income Country
NCSCT: National Centre for Smoking Cessation and Training
NTP: National Tuberculosis Control Programme
SSI: Semi-Structured Interview
TB: Tuberculosis
