# Supplementary material for: Using behaviour change theory to train health workers on tobacco cessation support for tuberculosis patients: a mixed-methods study in Bangladesh, Nepal and Pakistan
Source: BMC Health Serv Res. 2019 Jan 25;19:71. doi: 10.1186/s12913-019-3909-4 (PMC6347762; doi:10.1186/s12913-019-3909-4)
Supplement: Supplementary file 1 — COM-B Questionnaire –English Version. English version of adapted COM-B Questionnaire (DOCX 35 kb) [file 12913_2019_3909_MOESM1_ESM.docx]

** Adapted NCSCT questionnaire (following piloting) for Health Professionals/DOTS facilitators to assess Motivation, Capability, Opportunity for Cessation** Translated and adapted to individual country context/language in Bangladesh, Nepal, and Pakistan.

Assessing health worker capability, opportunity and motivation to provide key behavior change techniques to patients (BCTs added)

**Respondent ID**: __________

**Have you received training from the TB&Tobacco project?: Yes/No**

**If yes: Course Location: __________ and Date: __________**

**Have you received any other training on tobacco cessation?: Yes/No**

**If yes: Course Location: __________ and Date: __________**

To help us evaluate your confidence to provide tobacco cessation support in your facility context, we would be grateful if you could complete both pages of this short questionnaire. All your responses will be kept confidential.

**QUESTIONS ON TOBACCO CESSATION DELIVERY**

For statements 1-14 please circle the number corresponding with how you feel from a scale of 1 ‘not confident’ to 5 ‘highly confident’

| **How confident are you in doing each of the following?** | | **Not confident** |  | **Moderately confident** |  | **Highly confident** |
| --- | --- | --- | --- | --- | --- | --- |
| 1 | Describing what to expect from the treatment programme, including length, content and what it requires  *(RC9 Patient interaction and communication)* | 1 | 2 | 3 | 4 | 5 |
| 2 | Building rapport and asking male patients about tobacco use.  *(RC1 Patient interaction and communication)* | 1 | 2 | 3 | 4 | 5 |
| 3 | Building rapport and asking female patients about tobacco use.  *(RC1 Patient interaction and communication)* | 1 | 2 | 3 | 4 | 5 |
| 4 | Describing the principles and effectiveness of typical behavioural support  *(RC9 Patient interaction and communication)* | 1 | 2 | 3 | 4 | 5 |
| 5 | Facilitating and advising on use of social support (from friends, relatives, colleagues or ‘buddies’)  *(A2 Promote adjuvant activities)* | 1 | 2 | 3 | 4 | 5 |
| 6 | Describing the stop smoking medications that can support a quit attempt  *(A1 Promote adjuvant activities)* | 1 | 2 | 3 | 4 | 5 |
| 7 | Assisting clients to set a quit date  *(BM10, BS4 Behaviour, skills and motivation)* | 1 | 2 | 3 | 4 | 5 |
| 8 | Enhancing clients’ motivation and self-efficacy  *(BM2 Behaviour and motivation)* | 1 | 2 | 3 | 4 | 5 |
| 9 | Emphasising the importance of the ‘not a puff’ rule  *(BM10 Behaviour and motivation)* | 1 | 2 | 3 | 4 | 5 |
| 10 | Securing commitment to the ‘not a puff’ rule following the quit date  *(BM6 Behaviour and motivation)* | 1 | 2 | 3 | 4 | 5 |
| 11 | Helping clients to develop strategies to cope with barriers, cues to smoke and relapse triggers  *(BS1,2 and 3 Behaviour, skills)* | 1 | 2 | 3 | 4 | 5 |
| 12 | Assessing clients’ experience of stop smoking medications, including usage, side effects and perceived benefits  *(A3 Adjuvant activities)* | 1 | 2 | 3 | 4 | 5 |
| 13 | Dealing with lapses to minimise the likelihood that they will lead to full ‘relapse’  *(BS2 Behaviour and skills)* | 1 | 2 | 3 | 4 | 5 |
| 14 | Assessing commitment, readiness and ability to quit  *(RC7 Interaction and communication)* | 1 | 2 | 3 | 4 | 5 |
