# Supplementary material for: Using behaviour change theory to train health workers on tobacco cessation support for tuberculosis patients: a mixed-methods study in Bangladesh, Nepal and Pakistan
Source: BMC Health Serv Res. 2019 Jan 25;19:71. doi: 10.1186/s12913-019-3909-4 (PMC6347762; doi:10.1186/s12913-019-3909-4)
Supplement: Supplementary file 2 — Health Worker Semi-Structured Interview Guide. English version of health worker SSI guide. (PDF 47 kb) [file 12913_2019_3909_MOESM2_ESM.pdf]

Project Title: Tobacco Cessation within TB Programmes – Development and Feasibility (WP1)

Health Worker Semi-Structured Interview Guide

Welcome and thank you for making time for this interview.

Purpose and Format of the Interview:

As you may be aware, we are conducting an official study in collaboration with the National TB Programme and T&T on providing support for people with Tuberculosis (TB) who smoke and wish to quit smoking. We would like to ask you some questions about tobacco cessation and TB services in your facility, and are particularly interested in your views on how best we can help TB patients in your facility quit smoking. We are appreciative of your giving us time and would request as thorough responses as possible.

Challenges to Implementing TB Services in the Facility

1. Could you tell me about the services you provide to TB patients in your facility, and your role in delivering these services?

2. What are the main successes and challenges of delivering TB services in your facility?

Patients and Tobacco

3. How common do you think it is for your patients to use tobacco? What forms of tobacco do they use? Are there any differences between men and women?

4. In your opinion, what are possible reasons patients might use tobacco?

5. Do you ever ask patients about their tobacco use? How easy do they find it to talk to you about using tobacco? Does this differ between men and women?

6. What do you currently do if one of your patients says they use tobacco? {Probe into process of engagement with patient, steps taken, etc}

7. How do you think TB patients would respond to being offered support to quit tobacco use? Do patients face any challenges in admitting that they use tobacco?

8. What misconceptions to you think patients have about tobacco (all forms), and any links with TB?

9. How do you think patients will react to being offered a drug to help them quit? (Take into account the are already taking drugs for TB)

Delivery of Tobacco Cessation Support

10. {Describe the proposed intervention – gain any feedback on materials} How would you recommend integrating this intervention effectively within the routine working of the TB department in your facility?

11. How feasible would it be to give the cessation drug to patients? What facility or difficulty might be encountered? How would you manage storing the cessation drug and giving it to patients?

12. Realistically, how long do you normally have available to counsel a patient to quit tobacco use?

13. Where would you do the counselling?

14. What factors need to be considered in regard to providing counselling to patients?

15. Do you have any recommendations on how best to support illiterate patients to quit tobacco? Or any differences in how you would deliver this to men or women?

Opportunity for and Delivery of Intervention

16. What do you think the facilitators and barriers are to delivering tobacco cessation programmes within the TB services in your facility? (Probe on availability of resources, training, monitoring, supervision, time availability of health professionals and the layout of facilities, prescribing/discussing the cessation drug with patients)

17. Is tobacco currently part of any routine recording, reporting, monitoring or supervision mechanisms? If not, do you think this would be useful and feasible addition?

Capability for Cessation

18. How confident do you feel offering support to help TB patients to quit tobacco use?

19. Do you think you/other DOTS facilitators/health professionals need any additional skills to deliver tobacco cessation? What is the best way of increasing skills in this area?

20. What support from the District/Central TB programme/MoH would you/other staff in the facility need in order to provide tobacco cessation to TB patients?

21. From your experience as a health professional, do you have any recommendations on how best to build rapport and communicate well with patients?

Motivation for Cessation

22. How valuable/important do you think it would be to add tobacco cessation to routine TB services?

23. Do you feel your current workload would allow you to offer tobacco cessation to your patients?

24. How common is it for health professionals to use tobacco? Tell me about any experiences you have on this or any policies about this in your facility? Do you think this could have any implication in terms of delivering the intervention?

25. Is there anything else you would like to say about how the tobacco cessation intervention should be delivered in facilities like yours?

Get feedback on the key points and clarify anything you are unsure of.

Thank you for your time today. This is the end of the discussion. I will now turn off the recorder.
