# Supplementary material for: Using behaviour change theory to train health workers on tobacco cessation support for tuberculosis patients: a mixed-methods study in Bangladesh, Nepal and Pakistan
Source: BMC Health Serv Res. 2019 Jan 25;19:71. doi: 10.1186/s12913-019-3909-4 (PMC6347762; doi:10.1186/s12913-019-3909-4)
Supplement: Supplementary file 3 — Facility In-Charge Semi-Structured Interview Guide. English version of facility in-charge SSI guide. (PDF 47 kb) [file 12913_2019_3909_MOESM3_ESM.pdf]

Project Title: Tobacco Cessation within TB Programmes – Development and Feasibility (WP1)

Facility In-Charge Semi-Structured Interview Guide

Welcome and thank you for making time for this interview.

Purpose and Format of the Interview:

As you may be aware, we are conducting an official study in collaboration with the National TB Programme and T&T on providing support for people with Tuberculosis (TB) who smoke and wish to quit smoking. We would like to ask you some questions about tobacco cessation and TB services in your facility, and are particularly interested in your views on how best we can help TB patients in your facility quit smoking. We are appreciative of your giving us time and would request as thorough responses as possible.

Challenges to Implementing TB Services at District and Facility Level

1. Could you please describe the TB services in your facility? What are the main successes and challenges of delivering TB services in your facility?

Health Professionals' Motivation

2. {This question will explore if interviewee grasps the link between smoking and worse TB outcomes and motivation. If interviewee doesn't identify this link in response to question 2(a), go to question 2(b), otherwise go to question 2(c)}
   a. How valuable do you think it would be to add tobacco cessation to routine TB services in your facility? Why?
   b. {Explain the link between smoking and worse TB outcomes} In light of this fact, how valuable would it be to add tobacco cessation to routine TB services in your facility?
   c. Who do you think should be responsible for encouraging smoking cessation?

3. How common is it for health professionals to use tobacco? Tell me about any experiences you have on this or any policies about this in your facility?

Opportunity for and Delivery of Intervention

4. {Describe the proposed intervention – gain any feedback on materials} How would you recommend integrating this intervention effectively within the routine working of the TB department in your facility?

5. What do you think the facilitators and barriers are to providing tobacco cessation support effectively within the TB services in your facility? (Probe on availability of resources, training, monitoring, supervision, time availability of health professionals and the layout of facilities)

6. Are issues around tobacco (like tobacco use or passive smoking) currently part of any routine recording, reporting, monitoring or supervision mechanisms? If not, do you think this would be useful and feasible addition?

7. Do you feel your TB staff have sufficient time available to implement the intervention properly? (if possible probe on absenteeism) What obstacles might they face and how could these be addressed?

8. How would you organise the dispensing of the drug to be used in the trial?

Capabilities of Staff

9. Do you think DOTS facilitators/health professionals or DTLAs/DTLOs need any additional skills to deliver tobacco cessation? What is the best way of increasing skills in this area?

10. What support from the District/Central TB programme/MoH do you think staff in the facility need in order to provide tobacco cessation to TB patients?

11. How do you think TB patients would respond to being offered support to quit tobacco use? Do patients face any challenges in admitting that they use tobacco? Or in accessing this facility? Or other challenges?

Patients and Tobacco

12. From your experience as a clinician, do you have any recommendations on how best to build rapport and communicate well with patients?

13. Is there anything else you would like to say about how we should develop the tobacco cessation intervention in facilities like yours?

National Level Context of Tobacco Cessation

14. What do you think are the current priorities for the National TB programme?

15. Please can you tell me about any existing policies and programmes within Ministry of Health, including the National TB programme that have an element of tobacco cessation included within them?

16. Do you think there is momentum to address tobacco cessation within central government? If so, where has this come from? (probe on donor, civil society, media, research evidence or any other possible influences)

17. As far as you know, are there any financial resources available within the health system for tobacco cessation - in the TB programme, or others? Are any NGOs or donors implementing or supporting tobacco cessation programmes anywhere near here?

18. In your opinion, how do you think tobacco cessation programmes should be taken forward in our country's context?

Get feedback on the key points and clarify anything you are unsure of.

Thank you for your time today. This is the end of the discussion. I will now turn off the recorder.
