# Supplementary material for: Using behaviour change theory to train health workers on tobacco cessation support for tuberculosis patients: a mixed-methods study in Bangladesh, Nepal and Pakistan
Source: BMC Health Serv Res. 2019 Jan 25;19:71. doi: 10.1186/s12913-019-3909-4 (PMC6347762; doi:10.1186/s12913-019-3909-4)
Supplement: Supplementary file 4 — District-Level NTP Staff Semi-Structured Interview Guide. English version of district-level TB staff SSI guide (PDF 47 kb) [file 12913_2019_3909_MOESM4_ESM.pdf]

Project Title: Tobacco Cessation within TB Programmes – Development and Feasibility (WP1)

District-Level NTP Staff Semi-Structured Interview Guide

Welcome and thank you for making time for this interview.

Purpose and Format of the Interview:

As you may be aware, we are conducting an official study in collaboration with the National TB Programme and T&T on providing support for people with Tuberculosis (TB) who smoke and wish to quit smoking. We would like to ask you some questions about tobacco cessation and TB services, and are particularly interested in your views on how best we can help TB patients in your district quit smoking. We are appreciative of your giving us time and would request as thorough responses as possible.

National Level Commitment to Tobacco Cessation

1. What do you think are the current priorities for the National TB programme?

2. Could you please tell me about the wider context of tobacco control in the country and about any existing policies and programmes within Ministry of Health, including the National TB programme, that have an element of tobacco cessation included within them? (If appropriate, ask about recording/reporting mechanisms, or supervision and monitoring. This will be addressed again in questions 11-12).

3. Do you think there is momentum to address tobacco cessation within central government? If so, where has this come from? (probe on donor, civil society, media, research evidence or any other possible influences)

4. Do you think there are any obstacles to address tobacco cessation within the central government? If so, what are they? (probe on pressure or inducement from tobacco company employees)

5. Do you think other forms of tobacco (hookah, chewing tobacco) should be addressed within the TB programme?

6. What is the involvement of your organisation (if any) on tobacco?

7. Are there any MoHP/NTP programmes which you believe could and should have an element of tobacco cessation included within them? (Follow-up: How can existing tobacco cessation programmes be strengthened within MoH/NTP/etc? Should other actors be invloved?)

8. As far as you know, are there any financial resources available within the health system for tobacco cessation - in the TB programme, or others? Are any NGOs or donors implementing or supporting tobacco cessation programmes anywhere in the country or district?

Challenges to Implementing Intervention at District and Facility Level

9. Tell me about the TB programme in the district, how are services currently organised? What are the main successes and challenges within the district TB programme?

10. {Explore if interviewee grasps the link between smoking and worse TB outcomes and motivation. If interviewee doesn't identify this link in response to question 10(a), go to question 10(b), otherwise go to question 11}
    a. How valuable do you think it would be to add tobacco cessation to routine TB services? Why?
    b. {Explain the link between smoking and worse TB outcomes} In light of this fact, how valuable would it be to add tobacco cessation to routine TB services?

11. What do you think the facilitators and barriers are to delivering tobacco cessation programmes effectively within the TB programme in the district? (Probe on availability of resources, training, monitoring, supervision, time availability of health professionals and the layout of facilities)

12. Is tobacco part of any routine recording, reporting, monitoring or supervision mechanisms? If not, do you think this would be useful and feasible addition?

13. At what level of facility do you think tobacco cessation services should be offered? (e.g. district hospital, PHC, or community clinic/health post)

14. What do you think is the best way forward to integrate smoking cessation into the health system and TB programmes in particular?

15. Do you think tobacco cessation should be offered to only TB patients? Is it feasible to offer it to other patients?

16. Do you think DOTS facilitators/health professionals or DTLAs/DTLOs need any additional skills to deliver smoking cessation? What is the best way of increasing skills in this area?

17. What future plans are there for tobacco cessation in terms of policy making and implementation?

18. Is there anything else you would like to say about tobacco cessation?

Get feedback on the key points and clarify anything you are unsure of.

Thank you for your time today. This is the end of the discussion. I will now turn off the recorder.
